# Stochastic Micro-Pattern for Automated Correlative Fluorescence - Scanning Electron Microscopy

**DOI:** 10.1038/srep17973

**Published:** 2015-12-09

**Authors:** Isabell Begemann, Abhiyan Viplav, Christiane Rasch, Milos Galic

**Affiliations:** 1 DFG Cluster of Excellence ‘Cells in Motion’, (EXC 1003); 2 Institute of Medical Physics and Biophysics, University of Münster, Germany

## Abstract

Studies of cellular surface features gain from correlative approaches, where live cell information acquired by fluorescence light microscopy is complemented by ultrastructural information from scanning electron micrographs. Current approaches to spatially align fluorescence images with scanning electron micrographs are technically challenging and often cost or time-intensive. Relying exclusively on open-source software and equipment available in a standard lab, we have developed a method for rapid, software-assisted alignment of fluorescence images with the corresponding scanning electron micrographs via a stochastic gold micro-pattern. Here, we provide detailed instructions for micro-pattern production and image processing, troubleshooting for critical intermediate steps, and examples of membrane ultra-structures aligned with the fluorescence signal of proteins enriched at such sites. Together, the presented method for correlative fluorescence – scanning electron microscopy is versatile, robust and easily integrated into existing workflows, permitting image alignment with accuracy comparable to existing approaches with negligible investment of time or capital.

A powerful approach to gain insights into a biological process is to combine fluorescence live cell measurements with ultrastructural information from the same region. To acquire this type of data requires a hybrid approach called correlative light-electron microscopy (CLEM), where cells are dually imaged by fluorescence light and electron microscopy. Among the biggest challenges in CLEM are to find and to precisely align the same cell in the corresponding fluorescence and electron images. To solve these problems, a number of different protocols and devices have been developed over the past decades that allow either correlating light microscopy images with transmission or with scanning electron micrographs^1^,^2^,^3^,^4^,^5^,^6^. For samples where cross-sections of cells (i.e. TEM) are used to investigate events inside the cell, photo-oxidation techniques, grids and fluorescent beads have provided suitable tools to create coordinate systems for image correlation^7^,^8^,^9^. In contrast, alignment of fluorescence signal and surface topography (i.e. SEM), which is relevant to study biological processes at the plasma membrane^10^, has remained a challenging task. Current approaches rely either on sophisticated and often unique setups that permit simultaneous imaging of fluorescence and scanning electron microscopes^11^,^12^, or take advantage of micro-pattern that allow alignment of separately acquired fluorescence images and scanning electron micrographs^13^,^14^,^15^. A variety of strategies for image alignment, such as etched or deposited patterns for image alignment have been presented over the last years^13^,^14^,^15^,^16^,^17^. However, a precise and rapid alignment of fluorescent images and high magnification scanning electron micrographs has remained challenging using existing approaches, as these often rely on manual alignment of sparse and often repetitive patterns.

Here, we present a method that automatically aligns fluorescence and scanning electron images via a stochastic micro-pattern. These micro-patterns are rapidly generated in a one-step mask-free process, compatible with a variety of cell types, and provide sufficient spatial reference points for precise and robust software-assisted alignment of electron and fluorescence images even with large disparity in resolution.

## Results

### Generation of biocompatible stochastic micro-patterns

**Figure 1:**
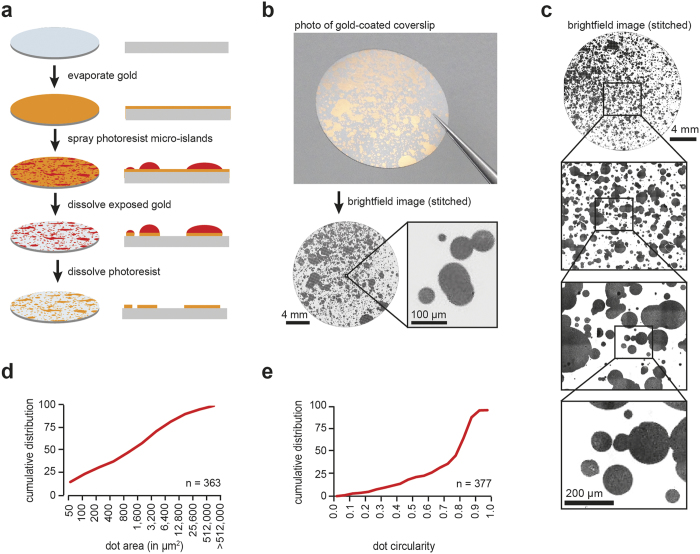
Generation of stochastic gold micro-pattern. **(a)** Flowchart of micro-pattern generation. Glass coverslips were coated with gold, and then sprayed with photoresist. Gold-coated areas that were not protected by photo-resist were dissolved by acid, and the protective photoresist layer was dissolved with DMSO, generating the gold micro-pattern. **(b)** Gold micro-pattern on a 18 mm circular glass coverslip is shown as a picture (top) and as stitched ‘global map’ composed of multiple bright-field images (bottom). Example of a single bright-field image is shown to the bottom right. **(c)** Zoom-in into a bright-field ‘global map’ of a 18 mm glass coverslip with gold micro-pattern. The pixel resolution of the global map is 325 nm. **(d)** Cumulative distribution of gold micro-pattern size shows a distribution of 50 μm^2^–500,000 μm^2^ in size. **(e)** Cumulative distribution of gold micro-pattern show high circularity. Scale bars **(b)**, 4 mm and 100 μm; **(c)**, 4 mm and 200 μm.

To create unique micro-patterns, glass coverslips were first uniformly coated with gold and then sprayed sub-confluently with photoresist (Fig. 1a–c, see Methods). Deposition of individual photoresist droplets on the gold substrate generated a random pattern of separate micro-islands (Supplementary Fig. 1a,b). Gold that was not protected by photoresist was then dissolved in an acid bath, followed by removal of the photoresist by DMSO. As photo-resist droplets varied in volume, this led to the formation of a stochastic pattern of gold micro-islands ranging in area from below 50 μm^2^ to over 500,000 μm^2^ (Fig. 1d, Supplementary Movie 1). While the smaller islands were spherical in shape, larger islands often show meandering boundaries due to the overlap with other droplets, thus adding landmarks that can be used for coarse orientation on the glass coverslip (Fig. 1b,c).

**Figure 2:**
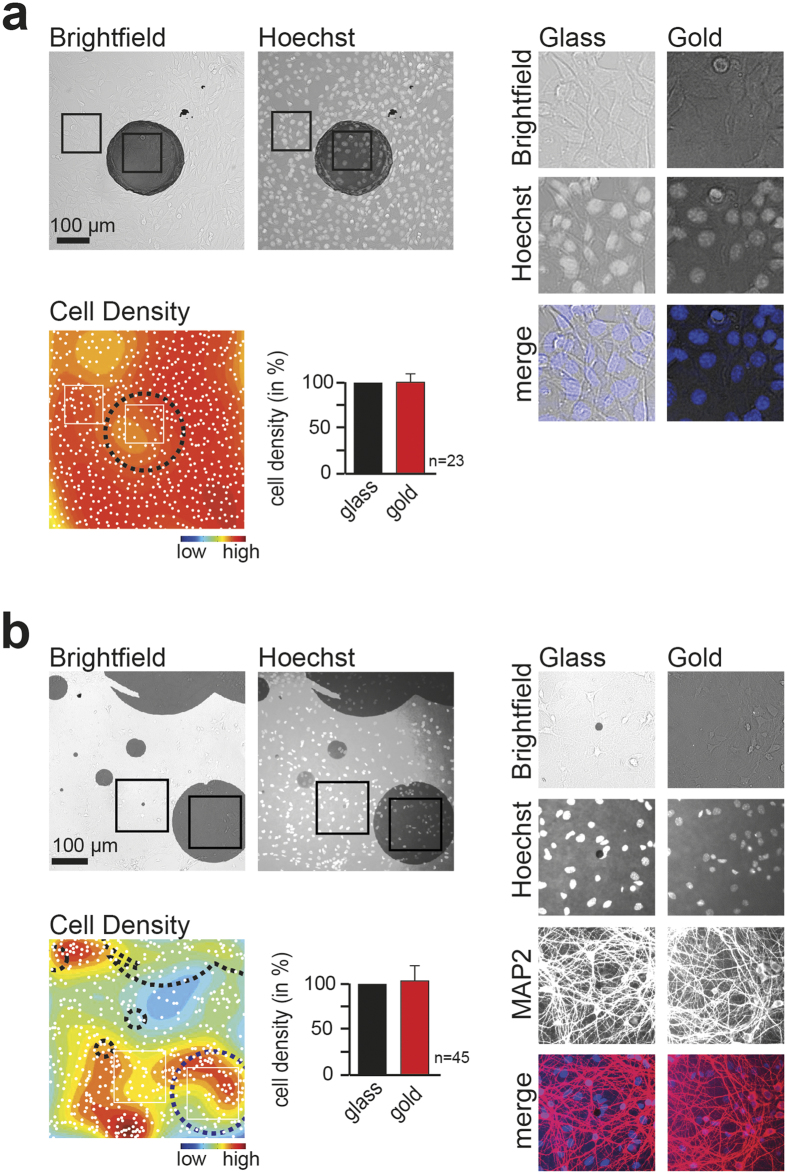
Stochastic gold micro-pattern is biocompatible. **(a)** 3T3 fibroblasts plated on micro-pattern show no preference for glass (light gray) versus gold substrate (dark gray). 3T3 fibroblasts were plated and cultured on micro-pattern, fixed and DNA was stained with Hoechst (blue). Bright-field image (top, left) and Hoechst staining (top, middle) as well as magnification of cells on glass and gold (top, right panels) are shown. Color-coded cell density map and quantification of 3T3 fibroblasts on gold and glass are shown below. **(b)** Primary hippocampal neurons plated on micro-pattern show no preference for glass (light gray) versus gold substrate (dark gray). Neurons were cultured for 8 days on the micro-pattern, fixed and stained with antibodies directed against the dendritic marker MAP2 (red) and Hoechst (blue). Bright-field image (top, left) and Hoechst staining (top, middle) as well as magnification of neurons on glass and gold (top, right panels) are shown. Color-coded cell density map and quantification are shown below. Note that neurons tend to cluster resulting in non-uniform global cell density. Scale bars **(a,b)**, 100 μm.

To test biocompatibility of the micro-pattern, we cultured cells on the gold substrate. We did not find a difference in the density of 3T3 fibroblasts on the gold compared to adjacent glass areas, arguing that the micro-pattern did not affect attachment or subsequent migration of cells (Fig. 2a). Likewise, we did not observe differences in the distribution of primary mouse hippocampal neurons that were cultured for 8 days on the gold micro-pattern (Fig. 2b). While we find that in both cases fluorescence intensity on the gold substrate is reduced, cells on the micro-islands are still clearly visible (Fig. 2a,b, right panels).

### Algorithms for automated mapping and alignment of fluorescence and electron microscopic images

**Figure 3:**
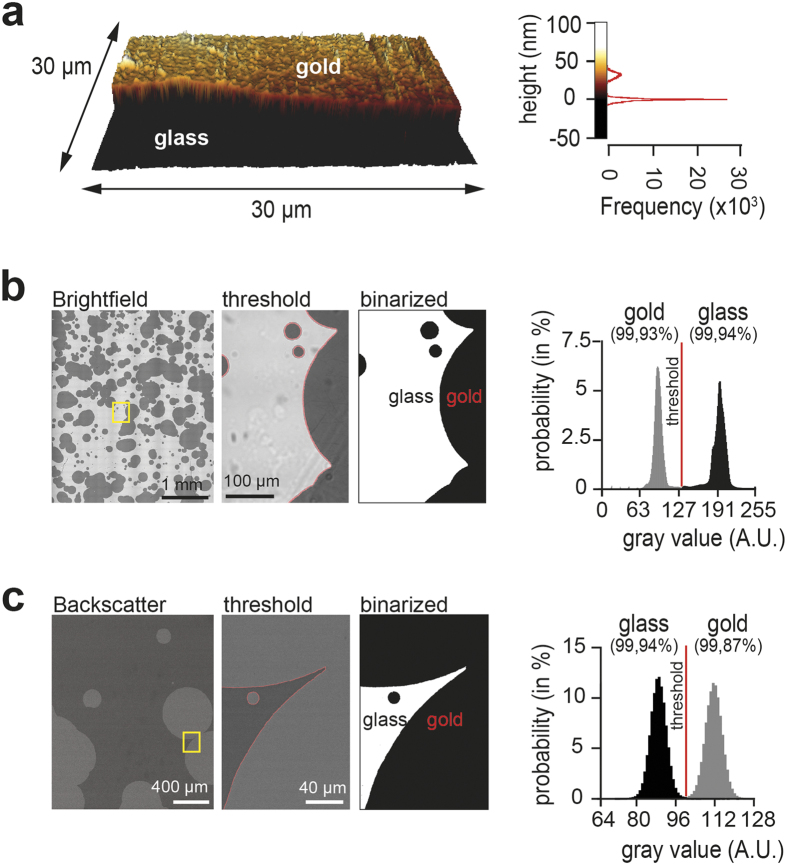
Detection of stochastic micro-pattern in light and electron microscopy. **(a)** Atomic force microscopic measurements show an average thickness of 30 ± 5 nm for gold. Raw height profile of a gold micro-pattern (left) and height frequency plot (right) are shown. **(b)** Differences in brightness allow a clear segmentation of glass vs. gold micro-pattern in bright-field. Quantification is shown to the right. Note that the gold layer is still transparent. **(c)** Differences in the number of back-scattered electrons allow a clear segmentation of glass vs. gold micro-patterns. Quantification is shown to the right. Scale bars **(a)**, 30 μm; **(b)**, 1 mm and 100 μm; **(c)**, 4 mm and 400 μm.

Gold micro-patterns of 30 ± 5 nm height (Fig. 3a) are clearly distinguishable from adjacent glass surface in bright-field (Fig. 3b, Supplementary Fig. 1c,d), as well as on the scanning electron microscope using the back-scatter detector (Fig. 3c). This is relevant, as it allows segmentation of gold and glass surface in images acquired on light and electron microscopes (Fig. 3b,c, right panels).

**Figure 4:**
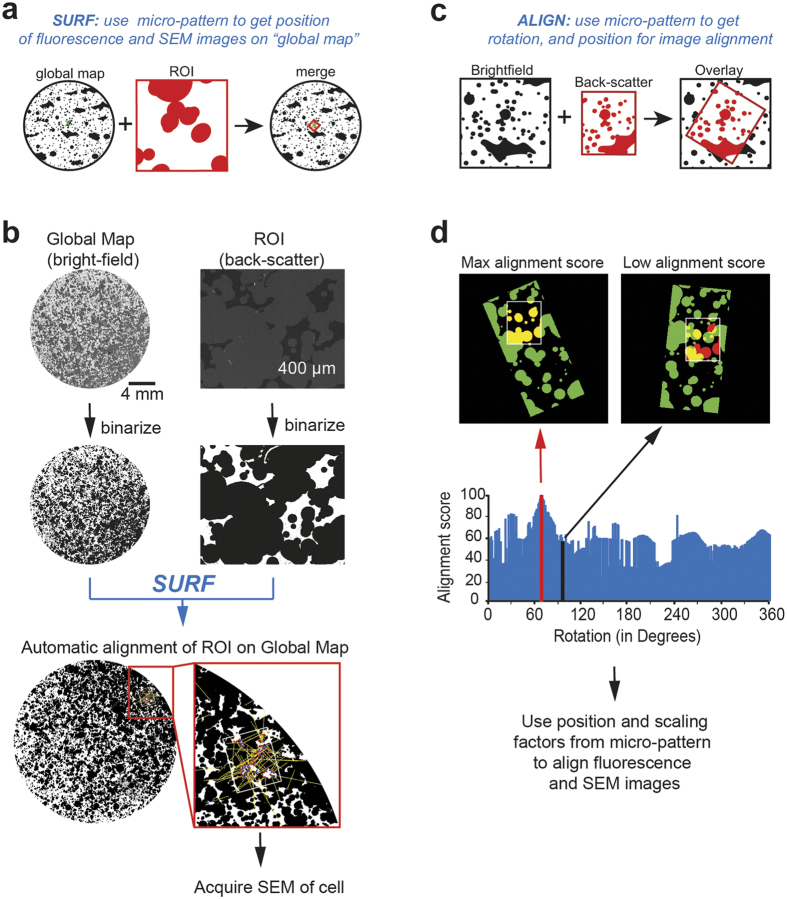
Automated alignment algorithms for CLEM via stochastic micro-patterning. **(a)** Gold-micro-patterns can be used to automatically find the precise localization of a cell on the global map via the SURF plugin. In a first step, for each fluorescence image a corresponding bright-field image is taken. Using the micro-pattern of the bright-field image, the position of the cell on the global map is defined (green cross). In a second step, the micro-pattern from the back-scatter detector (red, ROI) is used to navigate on the global map (black) and find the cell that was imaged with the light microscope (right panel). Finally, the SEM image of the cell can be taken. **(b)** Example of how the micro-pattern is used to find the position on the global map. The precise position of the micro-pattern from the back-scatter electron micrographs (right panels, i.e. ROI) on the global map of the whole glass coverslip (left panels, i.e. global map) is calculated using the SURF plugin (blue). Once the position is determined, the scanning electron micrograph of the cell imaged on the fluorescence microscope can be taken. Note that the SURF plugin was also used to annotate the precise coordinates of the cell on the global map (not shown). **(c)** Alignment of micro-pattern from electron and fluorescence images via the ALIGN macro. Schematic illustration of the problem of detecting position and rotation of two images for alignment is shown. **(d)** Example showing the ALIGN macro used to automatically align stochastic micro-pattern from bright-field (i.e. light microscope, green) and back-scatter (i.e. scanning electron microscope, red) images. Position and rotation from the micro-pattern are then used to align the representative fluorescence and scanning electron images of the cell. Scale bars **(b)**, 4 mm.

Correlation of fluorescence and electron micrographs requires in a first step to find with the scanning electron microscope the one cell that was previously imaged on the fluorescence microscope. To solve this task, we determined the relative position of the cell on the glass coverslip using the micro-pattern: For each cell we acquired in addition to the fluorescence image also a corresponding bright-field image depicting the micro-pattern surrounding the cell (i.e. ROI). Upon fixation of the sample, low magnification bright-field images of the entire glass coverslip were made and stitched together, creating a ‘global micro-pattern map’ (Fig. 1b,c, see Methods). Taking advantage of the SURF function, an ImageJ plugin, the position of the ROI (bright-field image showing the micro-pattern surrounding the cell) was automatically determined on the ‘global map’ (Fig. 4a). It took on average only 30 seconds to compute the coordinates of the ROI on the ‘global map’. Upon processing of the sample for SEM, micro-pattern from the back-scatter detector was used together with the SURF plugin to navigate to the exact same position on the coverslip (Fig. 4a,b). Notably, coordinate detection reliably worked independently of image magnification (Supplementary Fig. 2a), loss or damage of gold particles that may occur during SEM sample preparation (Supplementary Fig. 2b), or rotation of samples (Supplementary Fig. 2c), arguing that the alignment method is not only rapid, but also robust.

Once the same cell is identified and imaged with the fluorescence and scanning electron microscope, the corresponding images need to be aligned (Fig. 4c). Taking advantage of the micro-pattern present in the corresponding bright-field and back-scatter electron micrographs, position and rotation parameters for image fusion were automatically identified using a custom-made ImageJ macro, which we named ALIGN (Fig. 4d, Supplementary Fig. 2d, Supplementary Movie 2, see Methods). These parameters were then used to precisely fuse the corresponding fluorescence and scanning electron images, on which the micro-pattern are only faintly visible.

### CLEM of subcellular structures via stochastic micro-pattern

**Figure 5:**
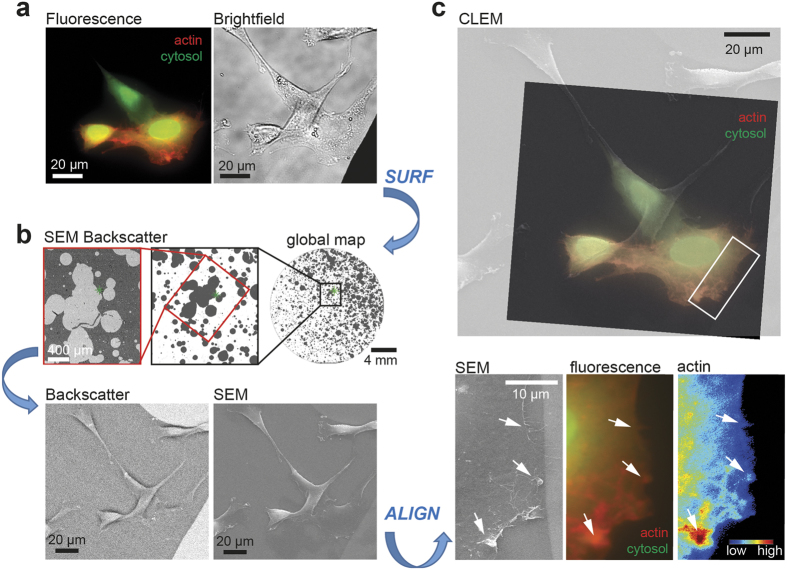
CLEM of subcellular structures via stochastic micro-pattern. **(a)** Alignment of electron and fluorescence images of 3T3 fibroblasts cultured on gold micro-patterns. 3T3 fibroblasts were cultured on the micro-pattern and transfected with a cytosolic fluorescence marker (green) and a marker for filamentous actin (red) 14 hours before fixation. Fluorescence image (left) as well as the bright-field image with the micro-pattern (right) was taken. **(b)** Using the SURF plugin with the back-scatter detector (red box), the cell (green cross) is identified on the SEM. Back-scatter and the corresponding SEM image are taken for the same region. **(c)** Alignment of fluorescence image and electron micrograph. Position and rotation for image alignment are automatically determined merging bright-field and back-scatter images with the ALIGN macro. These parameters are then used to merge the corresponding fluorescence and SEM images (top panel). Below, actin-rich structures at the leading edge (white arrows) are shown for high resolution SEM (left), fluorescence (middle) and actin (right). Scale bars **(a)**, 20 μm; **(b)**, 4 mm (top right), 400 μm (top left) and 20 μm (bottom); **(c)**, 20 μm (top) and 10 μm (bottom).

Next, we aimed to test the method on cells. For this, we plated 3T3 fibroblasts on the stochastic micro-pattern and transfected them with f-tractin, a fluorescence reporter directed against filamentous actin^18^, and a cytosolic reference. Using the SURF plugin described above (Fig. 4a,b), we took advantage of the micro-pattern to acquire fluorescence and scanning electron micrographs of the same cell (Fig. 5a–c), thus showing that the presence of cultured cells did not interfere with image segmentation or analysis. Position and rotation parameters for merging of fluorescence image and scanning electron micrograph were then determined using the micro-pattern from bright-field and back-scatter images with the help of the ALIGN macro. In a final step, multiple high-resolution images that were acquired from subcellular regions of interest were automatically embedded in a low resolution SEM image of the whole cell (Supplementary Fig. 3, see Methods). Taking advantage of this ‘enhanced’ SEM image, creation of only one correlated light – electron micrograph was sufficient to visualize low magnification global cell features, as well as high-resolution sub-cellular structures like contracting actin-rich patches that create highly convoluted membrane ruffles^19^ at the leading edge (Fig. 5c).

**Figure 6:**
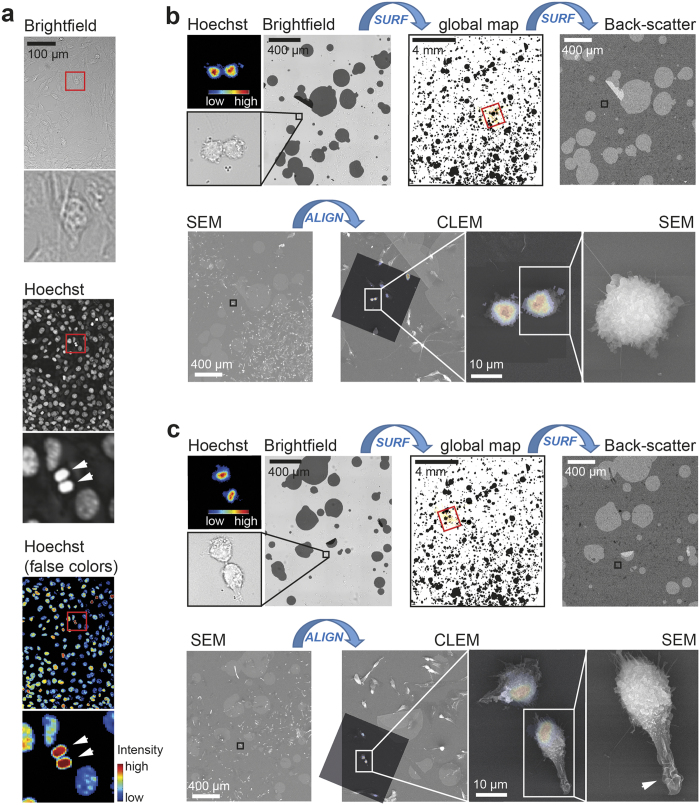
CLEM of 3T3 fibroblast in anaphase via stochastic micro-patterning. **(a)** Bright-field (top) and Hoechst staining (middle, bottom) of 3T3 fibroblasts. Note that 3T3 fibroblast in anaphase (white arrows) represent only 1% of all cells. **(b,c)** CLEM images of 3T3 fibroblasts during late anaphase. Individual cells were imaged with Hoechst and bright-field (top, left), and the relative position of the cell on the global map was identified with the SURF function (top, middle). Using the SURF function together with the back-scatter (top, right), the same cell was identified on the scanning electron microscope (bottom, left). Using micro-pattern from bright-field and back-scatter images with the align macro, position and coordinates for the CLEM alignment (bottom, middle) were determined. Note that cells during anaphase show a highly convoluted plasma membrane (bottom, right). Scale bars **(a)**, 100 μm; **(b,c)**, 400 μm, 4 mm and 10 μm.

Finally, we aimed to test whether the micro-pattern can also be used to align rare cellular events that spread across large areas. Specifically, we focused on investigating cell shape changes during anaphase, the separation of chromosomes during cell division^20^. As this phase is highly dynamic^21^, it accounts for only about 1% of the cell cycle (Fig. 6a). Notably, we were not only able to identify and align fluorescence and electron images from individual 3T3 fibroblasts during anaphase, but also to identify changes in membrane morphology (Fig. 6b,c) as well as initiation of cell polarization after cell division (Fig. 6c, arrow). Together, these results suggest that the method is not only fast and robust, but also scalable over several orders of magnitude.

### Analysis of robustness, efficiency and accuracy of micro-pattern-based image alignment

To determine whether gold particle loss may occur during cell culturing and subsequent sample preparation, a lift-off analysis was carried out. Specifically, we separately analyzed the stability of the gold micro-pattern during culturing cells and during processing the sample for SEM microscopy. We find a loss of 4.48 ± 2.00% and 0.80 ± 0.35%, respectively (Supplementary Fig. 4). Considering that such a loss can easily be intercepted by both, the ALIGN and the SURF function (see Supplementary Fig. 2b), gold lift-off through washing, cell culturing, fixation and SEM treatment processes is not an issue for image alignment.

Next, we aimed to investigate how alignment efficiency is affected by objective magnification, as well as the size and density of individual gold micro-pattern. As our approach creates a spectrum of particle diameters, we used *in silico* matrices with precisely controlled uniform dot diameters (Supplementary Fig. 5 and Methods) to probe the rate of successful image alignment (i.e. alignment efficiency). We find that alignment efficiency of small dot diameters is better with high magnification objectives, while large dot diameters work better with low magnification objectives (Supplementary Fig. 6). Notably, the matrix with randomly distributed dot sizes, mimicking the stochastic gold micro-patterning that we generate with the method, showed the best over-all alignment efficiency of all dot sizes that were tested (Supplementary Fig. 6, red panels). Taken together, stochastic micro-patterning with variable size distribution generated with our method provide not only landmarks for coarse orientation on the coverslip (via large micro-pattern) but also a reference point system for image alignment that unlike micro-pattern of uniform size can be scaled over several orders of magnitude while remaining highly efficient. As an added note, the observed decrease in alignment efficiency with large dot diameters can be explained with the reduced density of such objects. Consistently, an increase in dot density improves alignment efficiency (Supplementary Fig. 7).

Finally, we aimed to estimate alignment accuracy of our approach. For this, two overlapping images were cropped from the matrix with randomly distributed dot sizes. To avoid alignment artifacts due to a constant pixel-shift, lateral and horizontal shift varied for each cropped image pair, and 8 different scaling factors were used. The alignment error for each image was then determined by comparing the real and the calculated overlap position. We find an average alignment error of 0.396 ± 0.02 pixel (Supplementary Fig. 8a). Although our method applies diffraction-limited optical imaging, these measurements suggest that an accuracy of 80 nm (using a pixel-resolution of 200 nm, which is at the diffraction limit) can be achieved. Intriguingly, we find that alignment accuracy is limited by the image with lower resolution (Supplementary Fig. 8b), arguing that in our experimental setting alignment error is mainly determined by images taken by the light and not the electron microscope.

## Discussion

We describe the development and application of a new method for correlative fluorescence - scanning electron microscopy, which takes advantage of stochastic micro-pattern to automatically find and align corresponding images from light and electron micrographs. We find a uniform deposition thickness of 30 ± 5 nm gold to be optimal, as it allows clear segmentation of micro-patterns (Fig. 3a–c), while still being sufficiently transparent to permit imaging trough the gold layer (Fig. 2a,b, Supplementary Fig. S1d). We did not observe plasmonic enhancement^22^ in images of fluorescently labelled cells on the gold micro-pattern (Fig. 2a,b).

Using 30 nm thick gold micro-patterns that can be rapidly generated in a one-step mask-free process, we reliably achieve fast and automated alignment of sub-cellular structures (Figs 5,6). Previously described micro-pattern methodologies for identifying objects of interest in light and scanning electron microscopy rely on time consuming and expensive pattern design or mask production^23^. In comparison, our mask-free stochastic gold micro-pattern can be generated within ~45 minutes (Supplementary Fig. 9 and Supplementary Notes). The time required for finding the same region of interest (ROI) in fluorescence images and scanning electron micrographs is seconds, which is comparable with previously described methods using customized patterns or electron microscopy finder grids^23^,^24^. Although recent advancements using integrated microscopes circumvent the need for specimen transfer and subsequent ROI retrieval by simultaneous fluorescence and scanning electron imaging^11^,^25^,^26^, we believe that the proposed method is still of broad interest considering its cost effectiveness and compatibility with conventional light and electron microcopy setups present in laboratories.

To further advance alignment robustness, the number of reference points in the images would need to be increased, which could be achieved for example by creating a Voronoi^27^ polygon from the center of mass of individual gold micro-islands (Supplementary Fig. 10). As mechanical damage is likely to affect preferentially the edges of the individual micro-pattern (e.g. during processing of the sample), it is reasonable to conjecture that combining micro-patterns with Voronoi polygons will help to further improve the robustness of image alignment.

Alignment accuracy is defined by the objective magnification that is used and the pixel size of the camera. Assuming a pixel resolution of 200 nm, which is at the diffraction limit, our approach can achieve an alignment accuracy of ~80 nm, and is thus as good as previously described conventional correlative light-electron microscopy approaches^23^,^28^,^29^. Intriguingly, reports using resin sections show that fiducial landmarks are suitable to align florescent images and transmission electron micrographs with a precision of ~100 nm^30^, and this alignment precision could be further increased to 20–30 nm when combined with super-resolution fluorescence microscopy^17^,^31^. Considering that fluorescent markers can easily be cross-linked to gold^32^, one could envision that fluorescent labelling of our stochastic gold micro-pattern may render the approach suitable for STORM imaging, thus further improving alignment accuracy while still relying on freely available Image J plugins^33^,^34^. However, quality of correlative fluorescence – scanning electron micrographs does not only depend on image alignment, but also on the amount of unwanted movement of the sample that may occur due to osmotic or temperature changes during chemical fixation^35^,^36^,^37^. Thus, not only the micro-pattern but also the preservation-techniques would need further improvements to increase alignment accuracy. Considering the rapid immobilization of the samples achieved with cryo-fixation, high-pressure freezing may currently present the most promising strategy to solve this issue 38,39,40.

Importantly, we find that stochastic micro-patterning is scalable in both directions: the heterogeneity in the size of individual dots renders the approach not only suitable to investigate sub-cellular events, but also for applications where infrequent cellular events that spread across a large area need to be monitored (Fig. 6). This is significant as it argues that stochastic micro-pattern can be used to align structures over several orders of magnitude. Moreover, measurements using matrices containing randomly distributed dots suggest that the stochastic size distribution of gold micro-pattern increase efficiency compared to methods that rely exclusively on micro-pattern of one size (Supplementary Figs 5–8).

The presented method has been developed to correlate protein localization from fluorescence images with surface information derived from scanning electron micrographs. Intriguingly, recent work suggests that semi-thin resin sections stained with uranyl acetate and lead citrate, and imaged with the SEM backscatter electron detector can provide images similar to transmission electron micrographs of ultra-thin sections without the obstruction of grids, which are present in traditional TEM^41^. The study further applied semi-thin section of LR white resin-embedded specimens to correlate light and scanning electron microscopy using a FluoroNanogold-labeled secondary antibody. While beyond the scope of this work, these findings raise the possibility that the presented method may also be applied to cells sections.

In summary, we introduce a robust, scalable and rapid method for correlative fluorescence - scanning electron microscopy. The presented method provides an attractive alternative to existing approaches, as it is compatible with each microscopy setup, and relies on freely available software and micro-patterns that can be rapidly produced using tools present in each lab. Considering that all materials are biocompatible (Fig. 2) and can be combined with a variety of cell-specific coatings (e.g. poly-D-lysine, laminin, collagen fibronectin), this stochastic micro-pattern provides great experimental flexibility while minimizing processing time and cost without compromising the quality of the CLEM experiment.

## Methods

### Stochastic pattern generation

Glass coverslips were cleaned in a sonicator for 5 minutes in 99.5% acetone (Roth, 5025.6) followed by 5 minutes in 99.9% ethanol (AppliChem, 4H014516). Gold sputtering was accomplished using a Sputter-coater (Balzers Union, Sputtering Device 07 120) by applying 15 mA (2.5 kV) for 2 minutes in a chamber flooded with argon under 42 TORR. Positive photoresist was purchased from Conrad (POSITIV 20, No. 813923 - 62). Stochastic micro-patterns were generated by spraying a ~30% confluent film on a previously gold-coated glass coverslip. The non-coated gold areas were etched with a 1:3:2 ratio of 70% nitric acid (Sigma-Aldrich, 438073), 32% hydrochloric acid (Roth, X896.2) and ddH2O for 15 to 20 seconds. The etching-process was then stopped using cell culture medium (DMEM containing 4.5 g/L D-Glucose, GlutaMax-I, and pyruvate (LGC Standards GmbH [ATCC], ATCC 30-202), 10% Fetal bovine serum (FBS) (Biochrom AG, 1149C50615) and 1% penicillin/streptomycin (10,00 U/mL/10,000 μg/mL) (Biochrom AG, 12212), and glass coverslips with the micro-pattern were afterwards washed with 99.9% ethanol. Photoresist was processed following the manufacturer’s protocol, and subsequently dissolved using 99.5% DMSO (Sigma-Aldrich, D5879) for 5 minutes. The remaining gold micro-pattern was then washed in 99.9% ethanol to remove any DMSO and left for drying. The washing procedure was repeated twice to ensure no residual DMSO was left. Before culturing, the coverslips were sterilized with 99.9% ethanol and afterwards kept under UV-light for 15 minutes. A detailed description on time requirements and troubleshooting can be found in Supplementary Fig. 9 and Supplementary Notes.

### Atomic Force Microscopy

For thickness measurements (Fig. 3a), a homogenous gold layer was deposited with the same sputter-coater (Balzers Union, Sputtering Device 07 120), applying the same parameters (15 mA (2.5 kV) for 2 minutes, in a chamber flooded with argon under 42 TORR) as for creating the gold micro-patterns. The gold-coated coverslip was then left in a wet-chamber for 2 days, and dipped into Millipore water. Gold micro-pattern that floated off the glass surface was subsequently picked up with an ultra-planar Myca slips (Ted Pella, 1-800-237-3526), and used for accurate measurements of the gold thickness. The AFM images of the sputtered gold layer were done in touching-mode using commercial cantilevers (Nanosensors, Classic Tip) on a JPK NanoWizard II instrument. Height analysis was performed using JPK image processing software.

### Fluorescence Microscopy

Images were captured using a CMOS camera (Hamamatsu Orca Flash 4.0, Model C11440-22C), mounted on the side port of an inverted microscope (Eclipse Ti-RCD, Nikon). Images for stitching the ‘global map’ (Fig. 1b,c) were captured using a 20x oil-objective with a binning of 1 × 1 under bright-field conditions (Cool LED pE-100; 2516). All fluorescence images were taken using a 60x water-objective and a binning of 1 × 1, using a 561 nm DPSS laser (150 mW), a 488 nm diode laser (200 mW) and 405 nm laser (60 mW), respectively. According to the laser wavelengths, T561 filter cube or QUAD filter cube (for 488 nm and 405 nm) were used.

### Sample Preparation and Image Acquisition for Scanning Electron Microscopy

To remove residual paraformaldehyde (i.e. PFA; Ted Pella, 18505), pre-fixed samples were washed four times with 1× PBS (Gibco, 10010-023) containing 120 mM sucrose (Sigma, S7903), and fixed overnight in 2.5% glutaraldehyde (i.e. GA; Agar Scientific, R1011) in 1x PBS (Gibco, 10010-023) containing 120 mM sucrose. To remove GA from the sample, the sample was then washed three times with 1x PBS containing 4% sucrose (Gibco, 10010-023). Next, incubation with 1% osmium-tetraoxide (Roth, 7436.1) in 0.1 M PBS (after Sörensen) was done for 1 hour. For water reduction, the sample was then processed in a dilution series of ethanol (AppliChem, 4H014516) from 30%, 50%, 70%, 90% and 2 × 99.9% each for 20 minutes. Following critical point drying, the sample was mounted with LeitC (Plano, G3300) on an aluminum slide, left to gas out, and finally coated with 2.5 nm of platinum-carbonate under a 65° rotation in an evaporation chamber (Balzers Union, BAF 300). The scanning electron images and the back-scattered images were taken with a FE-SEM Hitachi S800. The accelerating voltage applied in the SEM measurements was 30 kV with a working distance of 15 mm. For obtaining the backscatter micrographs, a semiconductor detector of the type 113 back-scattered electron detector (GW Electronics) was used.

### Culturing and Transfection of 3T3 Fibroblasts

NIH 3T3 embryonic fibroblasts (Leibniz Institut DSMZ, ACC-59) were cultured on micro-patterned glass coverslips using DMEM containing 4.5 g/L D-Glucose, GlutaMax-I, and pyruvate (Gibco, 31966-021), 10% fetal bovine serum (Biochrom AG, L11-004) and 1% Pen/Strep (10,000 U/mL/10,000 μg/mL) (Biochrom AG, 12212). Transfection was accomplished with Lipofectamine 2000 (Life Technologies, 11668-027) following the manufacturer’s protocol. After 14 hours of expression, cells were fixed using 1x PBS (Gibco, 10010-023) containing 4% PFA (Ted Pella, 18505) and 120 mM sucrose (Sigma, S7903) for 20 minutes, stained and kept in 1x PBS (Gibco, 10010-023) containing 4% sucrose until SEM processing.

### Culturing and Immunostaining of Primary Hippocampal Neurons

Sterilized gold micro-patterned glass coverslips were functionalized with poly-D-lysine in hydrobromide solution (70 μg/ml; Sigma, P6407) for 1 hour and rinsed off with ddH2O. Mouse hippocampal neurons were prepared as previously described^42^. In brief, primary neurons prepared form E18 mice embryos were plated onto functionalized gold micro-patterned coverslips using NBM (Neurobasal Medium, Gibco, 21103-049), supplemented with B27 (Gibco, 17504-044), Pen/Strep (Biochrom AG, 12212) and 20 mM HEPES (Gibco, 15630). For immunostaining, cells were fixed 8 days after plating (DIV 8) in 1× PBS (Gibco, 10010-023) containing 4% Formaldehyde (Ted Pella, 18505) and 120 mM sucrose for 20 minutes at room temperature and quenched for 20 minutes with 100 mM NH_4_Cl (Carl Roth, K298.2). Then blocking and permeabilization was performed with 0.1% Triton-X100 (Sigma, T9284) in PBS containing 2.5% bovine serum albumin (Sigma; A9085.25G) for 15 minutes at room temperature. The cells were incubated for 1 hour at room temperature with mouse monoclonal anti-MAP2 antibody (1:250; Synaptic Systems, 188 011) and Hoechst 34580 (2 μg/ml; Life Technologies, H21486). Primary antibodies were detected using goat anti-mouse 488 (1:1000: Invitrogen, O-6380).

### Image Analysis and Troubleshooting

Image analysis was performed using exclusively FIJI/ImageJ^43^. Formation of the ‘global map’ (Fig. 1b,c) was made using the ‘Grid/Collection stitching’ plugin^44^. In our hands an overlap of 30% between individual bright-field images worked best for stitching the global map. Finding the ROI on the ‘global map’ (Fig. 4a,b) was done with the SURF plugin from Eugen Labun (http://labun.com/imagej-surf/). To improve computational time, pixel resolution (i.e. image size) of the ‘global map’ can be reduced. Note that for good performance, several complete gold micro-islands should be visible in the ROI. In cases where this is not the case, increase the imaged area around the cell. For the ALIGN macro (Fig. 4c,d; script available upon request), the two images are rotated against each other and for each angle the ‘pairwise stitching’ plugin^44^ was applied. Merging of SEM images with different resolutions for the ‘enhanced’ SEM image (Supplementary Fig. 3) was performed using the ‘pairwise stitching’ plugin^44^. Density measurements of cells on glass and gold (Fig. 2) were performed in ImageJ, measuring the cell density on gold micro-islands and the adjacent glass surface. Voronoi polygons (Supplementary Fig. 4) were generated using the Delaunay Voronoi plugin.

### Fluorescence Markers and Dyes

Filamentous actin was labelled using F-tractin^18^. As cytosolic reference, we used an empty pEGFP(N3) plasmid (Clontech). DNA was labelled with Hoechst 34580 (2 μg/ml; Life Technologies, H21486).

### Analysis of gold lift-off during sample processing

A global map of an untreated coverslip with gold micro-pattern was imaged with an inverted microscope in bright-field using a 20× objective and stitched as described above. The same coverslip was then washed, prepared, and NIH 3T3 cells were plated. Upon fixation, 19 random areas from the coverslip were imaged and the number of present gold micro-pattern were manually counted on each area and compared to the number of gold micro-pattern in the same area of the untreated coverslip (Supplementary Fig. 4a).

For testing the influence of processing for SEM (see above), gold micro-pattern with plated cells were prepared, imaged with an inverted microscope in bright-field using a 20× objective, and images were stitched into a global map. After SEM-processing of the coverslips, 24 random regions were imaged with a SEM using a magnification of 60x (Supplementary Fig. 4b). As before, the number of present gold micro-pattern was counted for each region. In both cases, scratches on the gold micro-pattern created by forceps during sample handling were not considered (see example in Supplementary Fig. 4a, image g).

### Generation of 10,000 × 10,000 pixel matrices

A matrix of 10,000 × 10,000 pixels was established in ImageJ in 8-bit for each separate image. The random dots were created with the ImageJ plugin ‘DrawRandomDots’ by W. Rasband (http://code.google.com/p/fiji-bi/source/browse/DrawRandomDots.txt?repo=imageja&name=macros), in which the dot size and the density can be changed. For creating different defined dot sizes, the same plugin was taken with different dot sizes multiple times on the same matrix. Afterwards, the image was binarized and measured by the plugin ‘AnalyseParticles’ to measure the total dot area (Supplementary Fig. 5). For changing the coverage area, the dot number was changed and total dot area was measured again using the ‘AnalyseParticles’ plugin (Supplementary Fig. 7).

### Analysis of alignment efficiency

The camera (ORCA-Flash 4.0 LT, Hamamatsu) that was used in the experiments has 2,048 × 2,048 pixel with a size of 6.5 × 6.5 μm. Thus, one pixel in the acquired image is reflective of 65 × 65 nm for a 100× objective, 108 × 108 nm for a 60× objective, 162.5 × 162.5 nm for a 40× objective, 325 × 325 nm for a 20× objective, 650 × 650 nm for a 10× objective, and 1,625 × 1,625 nm for a 4× objective. We considered the pixel size on the matrices with the randomly distributed micro-pattern that we generated to be 65 nm. Consequentially, a 20 pixel dot reflects a diameter of 1.3 μm. For each matrix, 16 pairs of images of 2,048 × 2,048 pixel size with a constant overlap of 90% were generated. Considering the diffraction limit of light, the image was then rescaled by a factor of 3.846 (one pixel = 250 nm). Next, constant single pixel noise was added (salt and pepper function in ImageJ), and the image was blurred. Finally, the image was binarized with a threshold of 100 and rescaled to the previous size of 2,048 × 2,048 pixels. All 8 matrices (dot size = 20, 50, 100, 200, 250, 500, 1,000 and randomly distributed) were processed as described above, and for each dot size 16 separate image pairs were merged using the ALIGN function, and the rate of successful alignments for a 100× objective was scored (Supplementary Fig. 6a, right panel). To simulate objectives with other magnifications (60×, 40×, 20×, 10×, 4×), the 16 image pairs from each of the 8 matrices (with various dot patterns) were rescaled with the corresponding scaling factor and analyzed as described above (Supplementary Fig. 6b–g, right panels).

### Analysis of alignment accuracy

To measure alignment accuracy, the matrix with random dot size was used. To avoid alignment artifacts due to a constant pixel-shift, the lateral and horizontal shift for each of the 16 image pairs was different, and 8 different scaling factors between 1 and 4 were randomly picked. Images were rescaled and treated as described above, and for each of the 112 image pairs the error in x and y was translated into the total deviation (i.e. √(x^2^ + y^2^), see Supplementary Fig. 8a).

### Statistics

P-values in all figures depict pair-wise comparisons and were evaluated using the student t-test, with two tails and two-sample unequal variance. Error bars in all images represent SEM of the mean value. **P < 0.01.

## Additional Information

**How to cite this article**: Begemann, I. *et al.* Stochastic Micro-Pattern for Automated Correlative Fluorescence - Scanning Electron Microscopy. *Sci. Rep.* **5**, 17973; doi: 10.1038/srep17973 (2015).

## Supplementary Material

Supplementary Movie 1

Supplementary Movie 2

Supplementary Information
