# Supplementary material for: Stochastic Micro-Pattern for Automated Correlative Fluorescence - Scanning Electron Microscopy
Source: Sci Rep. 2015 Dec 9;5:17973. doi: 10.1038/srep17973 (PMC4673610; doi:10.1038/srep17973)
Supplement: Supplementary Information [file srep17973-s3.pdf]

Supplementary Information for:

Stochastic Micro-Pattern for Automated Correlative Fluorescence - Scanning Electron Microscopy

Isabell Begemann[1,2,3], Abhiyan Viplav[1,2,3], Christiane Rasch[2] and Milos Galic[1,2]

Author Affiliation:

[1] DFG Cluster of Excellence 'Cells in Motion', (EXC 1003)

[2] Institute of Medical Physics and Biophysics, University of Münster, Germany

[3] Equal contribution

Corresponding author:

Correspondence to: galic@uni-muenster.de

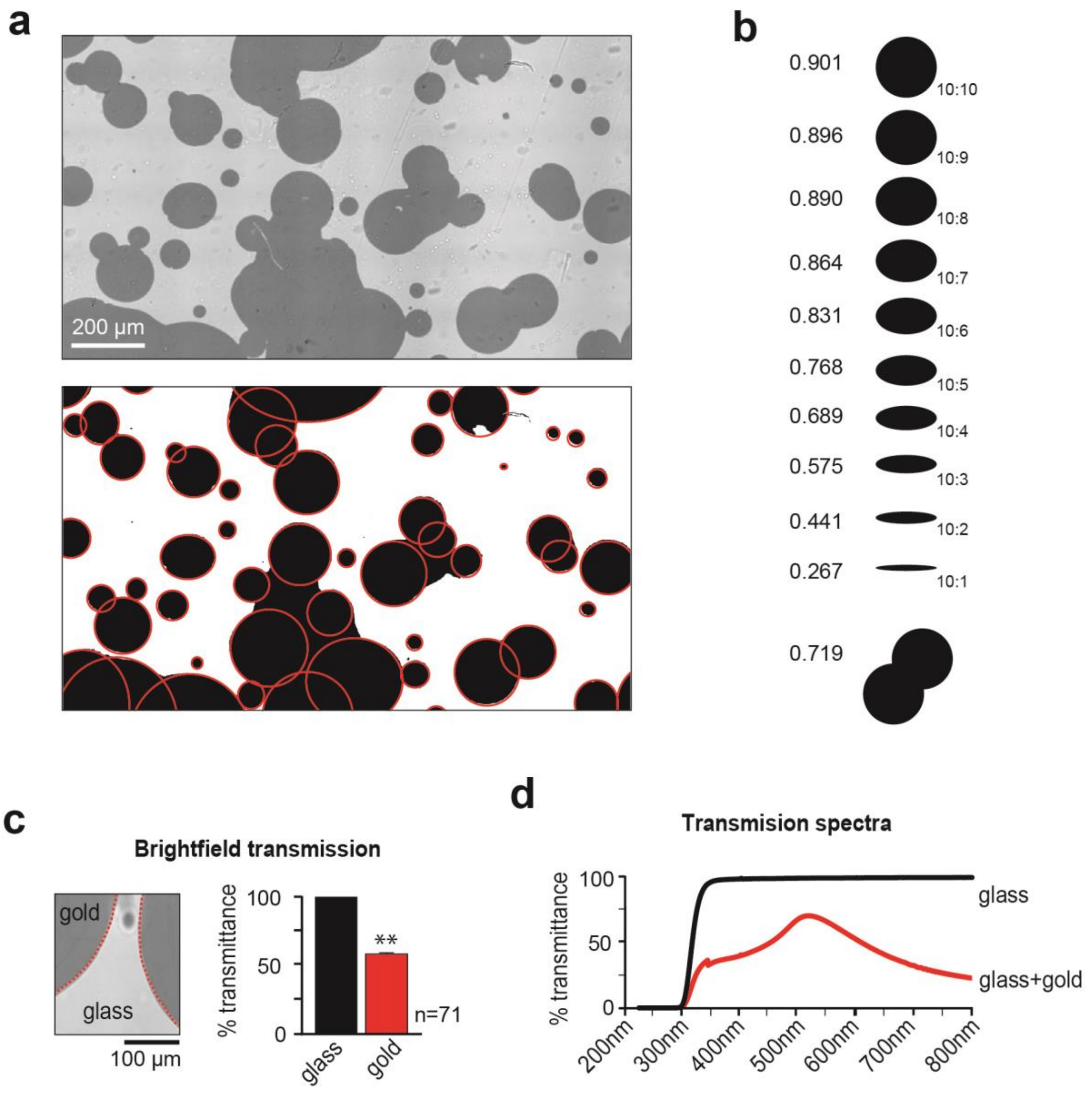

Supplementary Figure 1: Circularity of gold micro-pattern. (a) Stochastic gold micro-patterns can be depicted as an overlay of circles. **(b)** Examples of circularity measurement. **(c)** Average transmittance of glass and glass coated with 30 ± 5 nm thick gold layer on bright-field microscope. **(d)** Spectral transmittance of glass (black) and glass coated with 30 ± 5 nm thick gold layer (red). Scale bars, (a), 200 µm; (d), 100 µm.

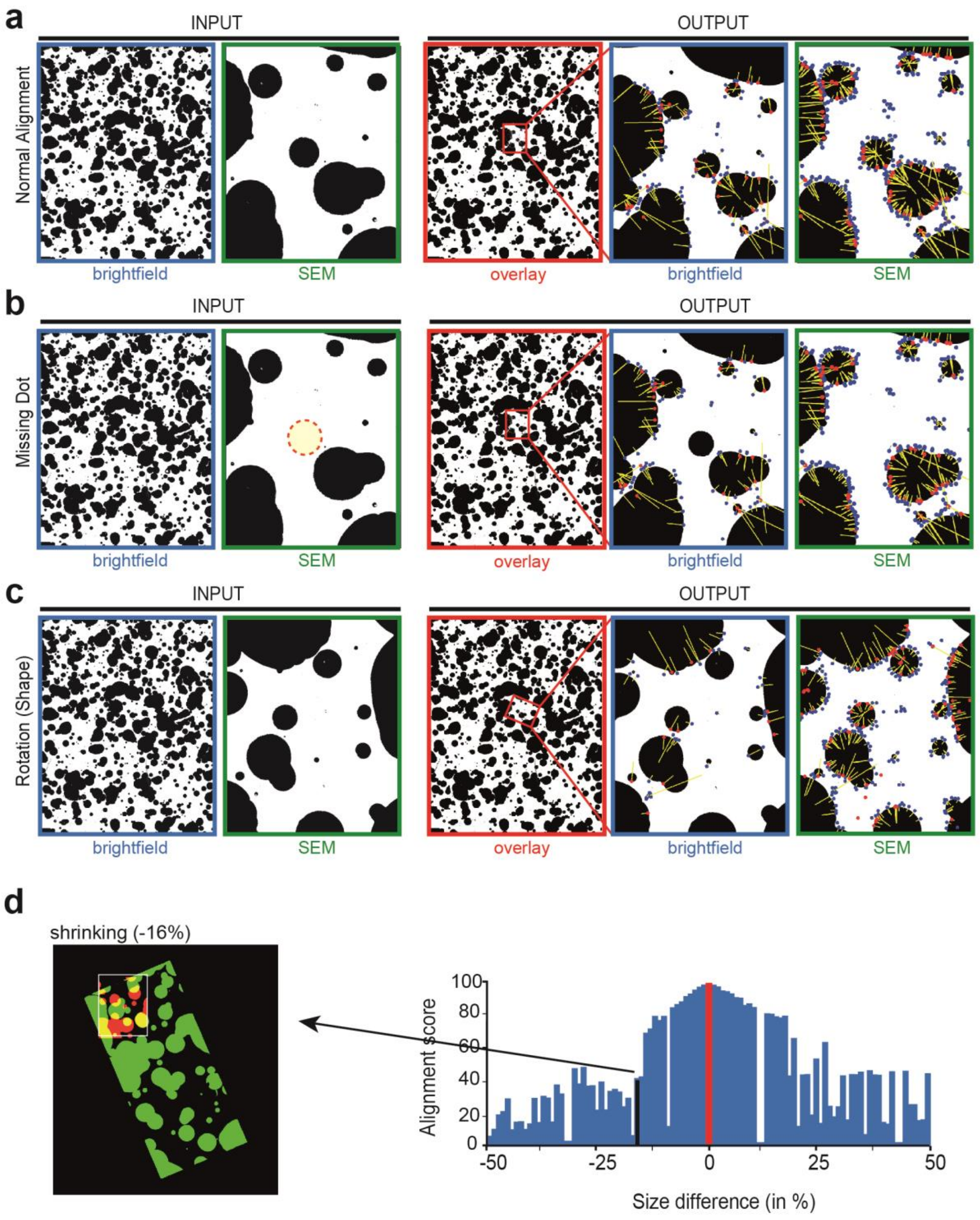

Supplementary Figure 2: Robustness of alignment algorithms. (a) ROI and global map that contain features of various pixel sizes (i.e. that differ in magnification) can be reliably aligned with the SURF plugin. **(b)** Alignment of ROI and global map with SURF plugin is robust to loss of individual gold micro-patterns. **(c)** Alignment of ROI and global map with SURF plugin is robust to rotation. **(d)** Binarized images of micro-pattern from bright-field (green) and back-scatter (red) images are scaled relative to each other to identify the precise size for image alignment. Alignment scores are shown next to the images.

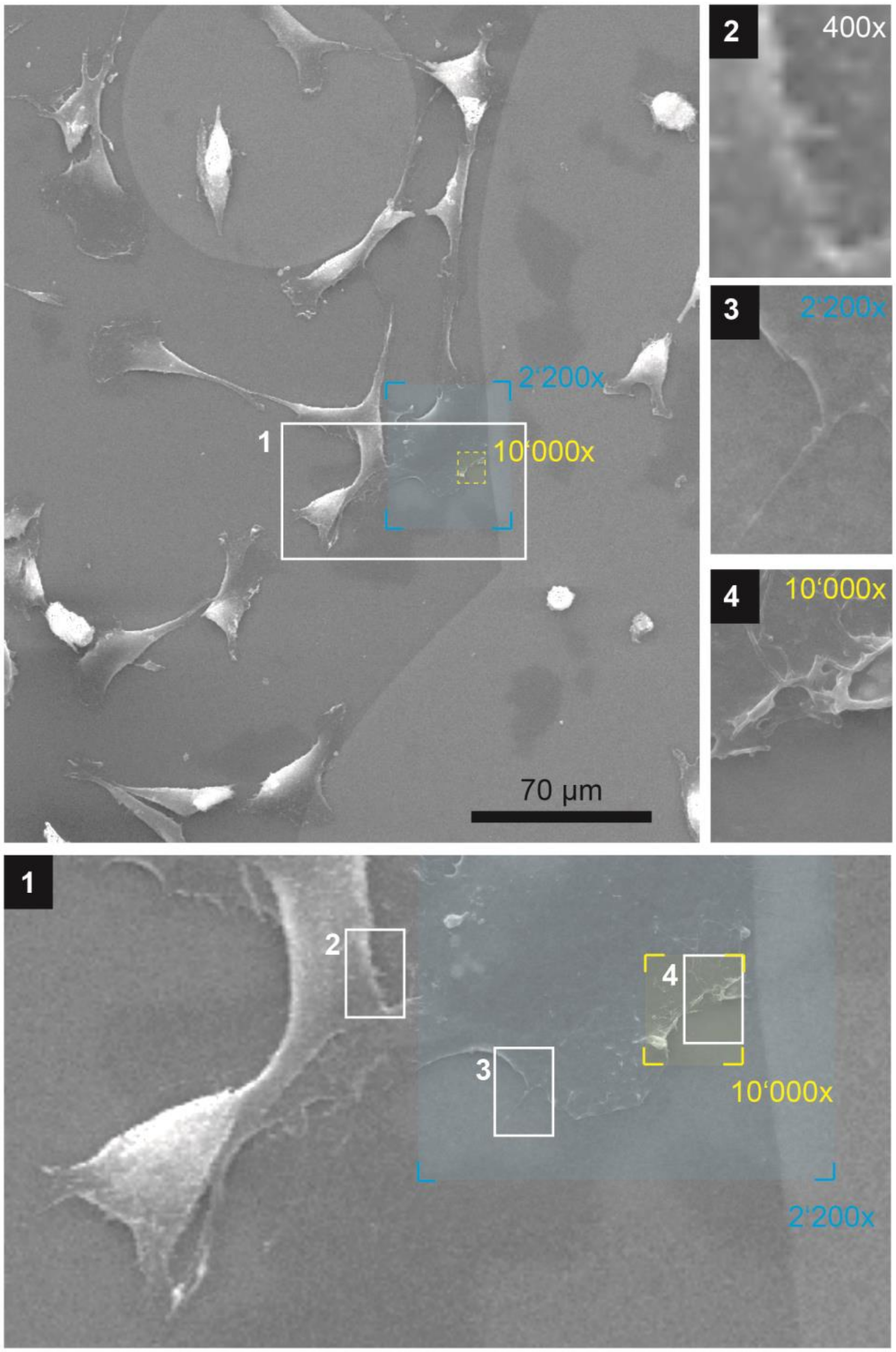

Supplementary Figure 3: Automatic fusion of high and low resolution scanning electron micrographs to create 'enhanced' SEM. Generation of an 'enhanced' SEM image via fusion of high-resolution (2,200x in blue, 10,000x in yellow) micrographs into a low resolution (400x) overview image using the ALIGN macro. For better illustration an overview (image 1) as well as images of the respective magnifications (images 2-4) is shown. Note the difference in resolution of cellular features between image 2 (875 pixels/100 µm2), image 3 (4,812 pixels/100 µm2) and image 4 (21,875 pixels/100 µm2). Scale bar,70 µm.

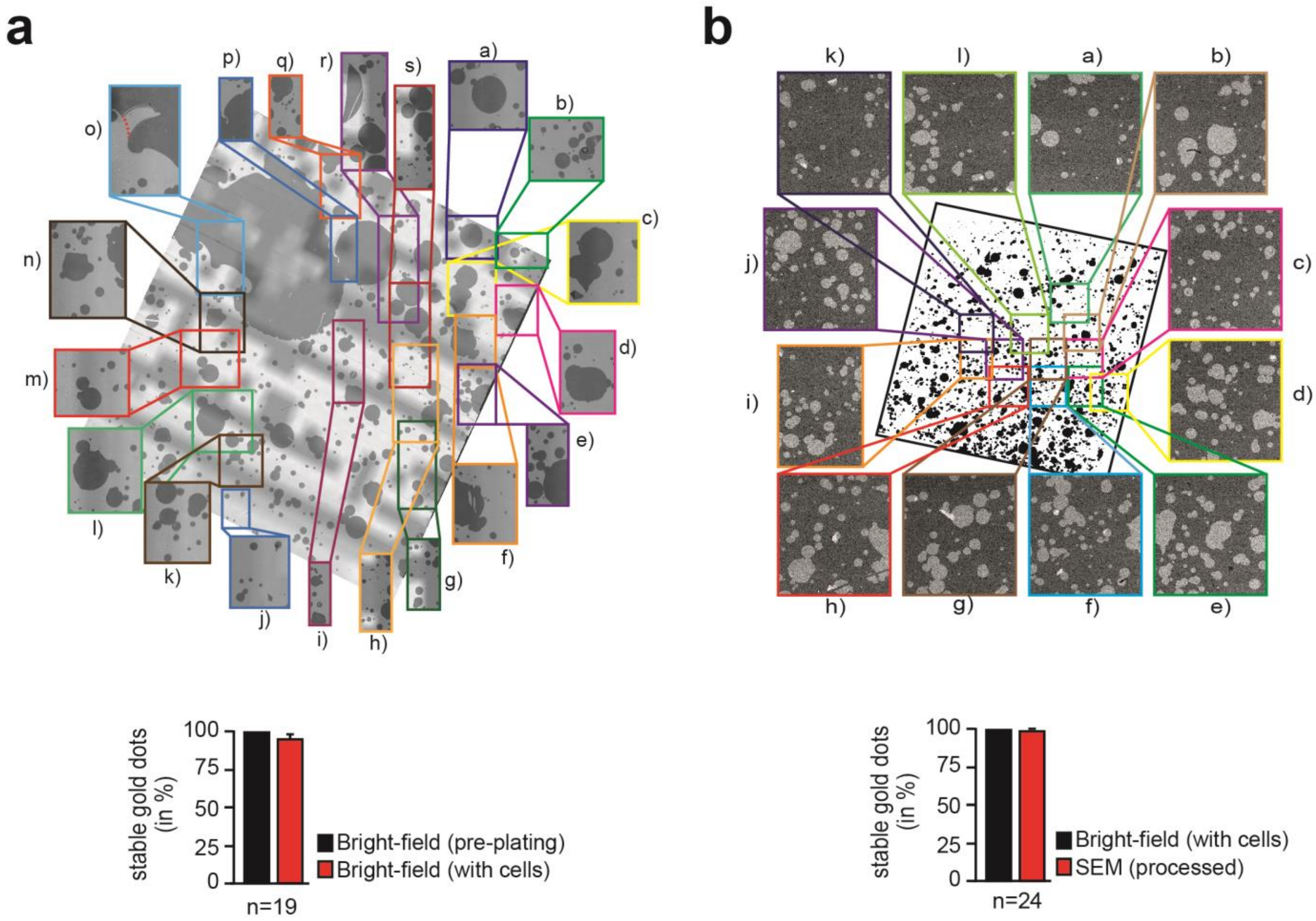

Supplementary Figure 4: Analysis of gold particle lift-off. (a) Overview of selected regions of the gold micro-pattern for lift-off analysis before and after processing for light microscopy. A global map of covers-lip with the untreated gold micro-pattern was stitched from 25 x 28 separate bright-field images taken with a 20x objective (center image). After several washing steps, NIH 3T3 cells were plated on the gold-coated coverslips and regions a) to s) were imaged using the same objective as before. Analysis of gold micro-pattern lift-off analysis is shown below. **(b)** Analysis of gold micro-pattern lift-off before and after processing for scanning electron microscopy. A global map of gold micro-pattern with plated cells was stitched from 29 x 29 separate images taken in bright-field with a 20x objective (center image). After preparation, samples were re-imaged using a SEM with a magnification of 60x. Analysis of 24 discrete regions from two coverslips is shown below.

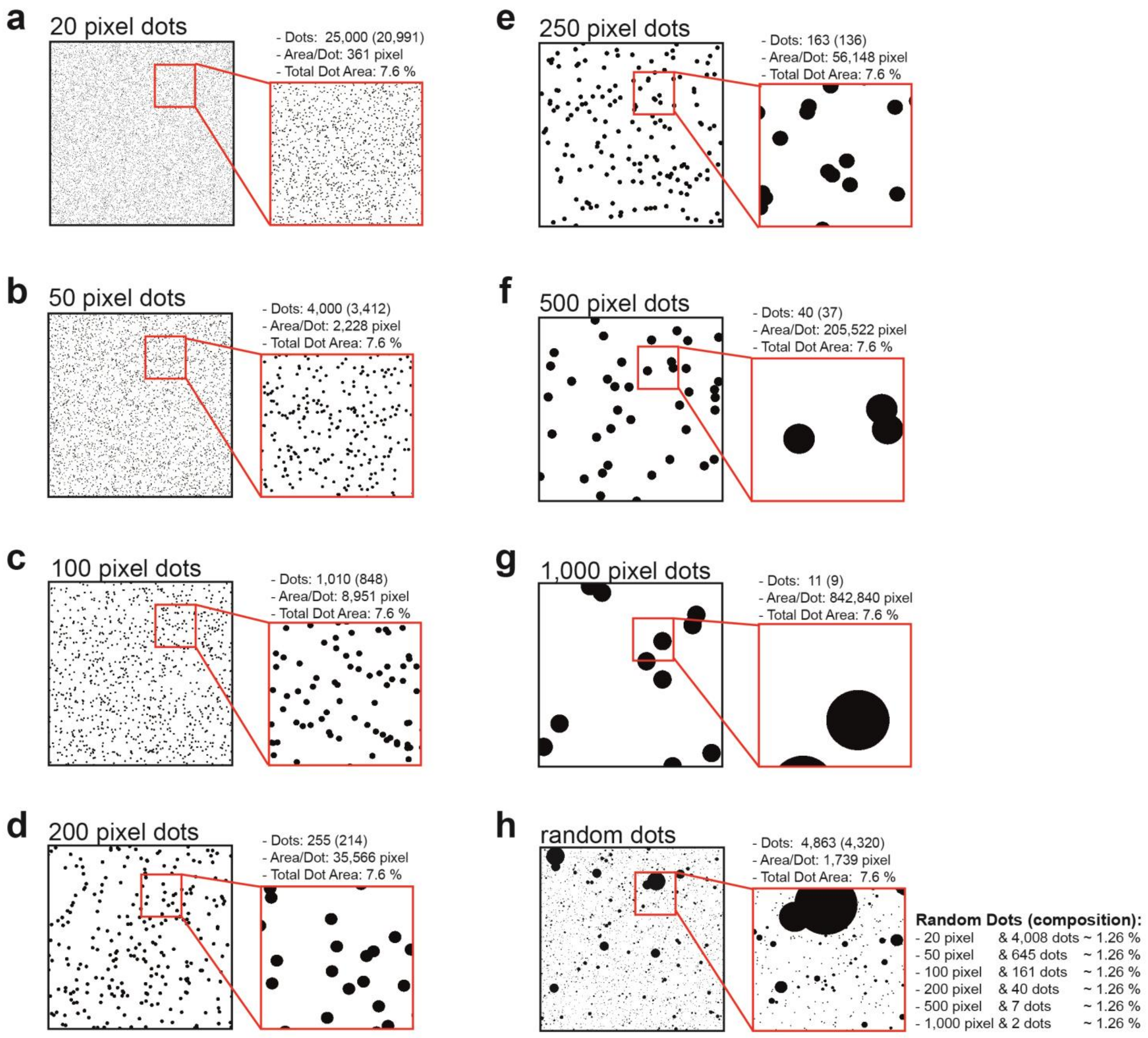

Supplementary Figure 5: ***In silico*** **generated micro-pattern with various size and constant densities.** To the left, matrices of 10,000 x 10,000 pixels containing micro-pattern with a definite diameter of 20 pixel **(a)**, 50 pixel **(b)**, 100 pixel **(c)**, 200 pixel **(d)**, 250 pixel **(e)**, 500 pixel **(f)**, 1,000 pixel **(g)** and a random distribution of diameters **(h).** All pattern cover in total 7.6 % of the whole matrix area. To the right, a zoom-in of the micro-pattern (a-h) is shown. For each matrix, the the number of created dots as well as the number of counted dots (in brackets), the average pixel-area per dot and the coverage in percent is shown. Note that the counted number of dots is smaller than the created ones due to partial overlap.

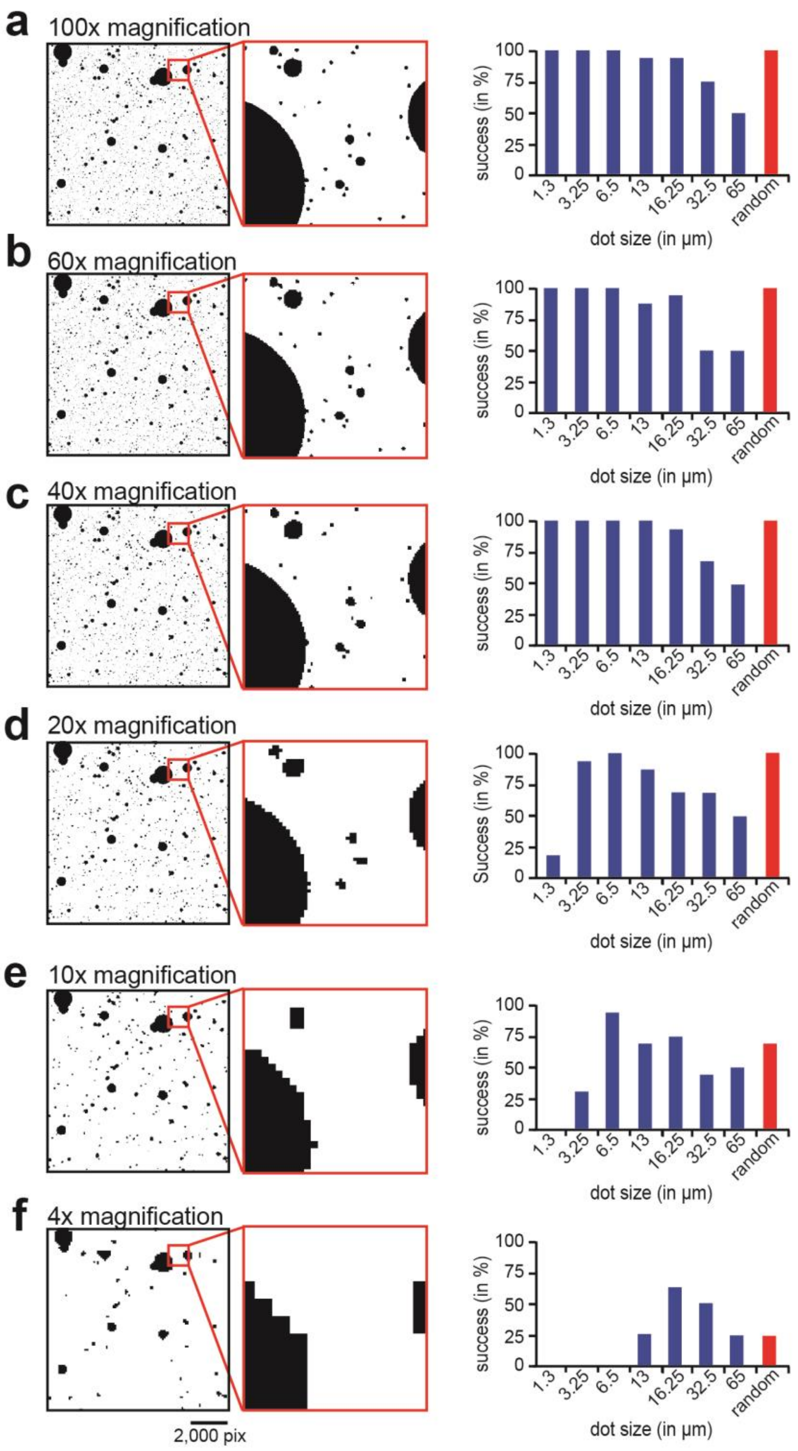

Supplementary Figure 6: Alignment efficiency of matrices with micro-pattern of different sizes. (a) Image of 10,000 x 10,000 matrix showing micro-pattern of random size as seen with a 100x objective is shown to the left. To the right, the percentage of successful alignment of 10,000 x 10,000 matrices containing micro-pattern with uniform diameters of 1.3 µm, 3.25 µm, 6.5 µm, 13 µm, 16.25 µm, 32.5 µm and 65 µm (all blue) as well as micro-pattern with random diameters (red) are analyzed. **(b-f)** To the left, image of 10,000 x 10,000 matrices showing micro-pattern of random size as seen with a 60x (b), 40x (c), 20x (d), 10x(e) and 4x (f) objective. To the right, analysis of alignment efficiencies for different matrices with the respective magnification is shown. Note that the random micro-pattern with random size distribution shows the best over-all alignment efficiency.

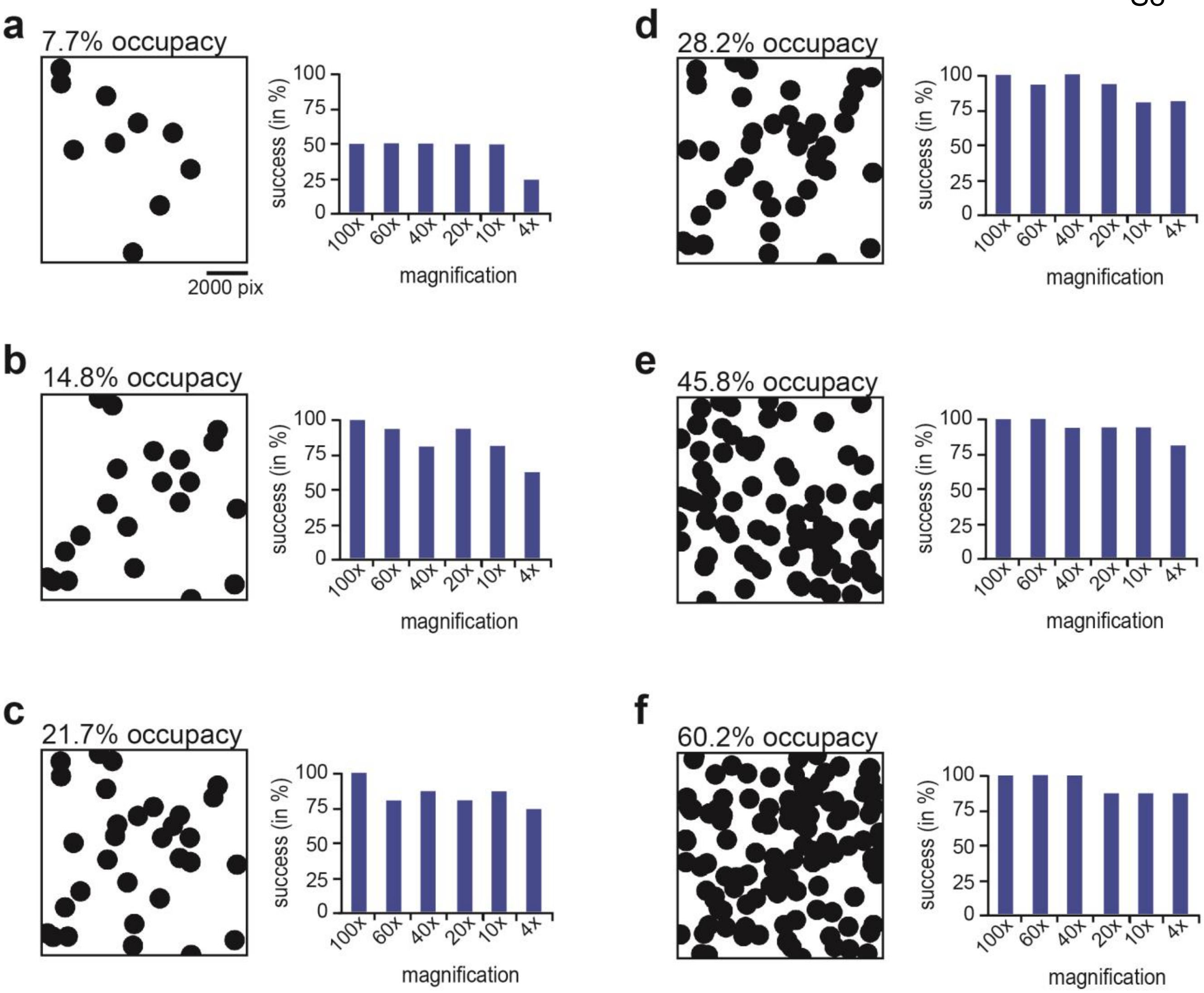

Supplementary Figure 7: Density-dependence of alignment efficiency. (a) Image of 10,000 x 10,000 matrix showing micro-pattern of 1,000 pixel size (as seen with a 100x objective) covering 7.7 % of the surface is shown to the left. To the right, percentage of successful alignment for the matrix at different magnifications is shown. **(b-f)** To the left, image of 10,000 x 10,000 matrices showing uniform micro-pattern size of 1,000 pixel (as seen with a 100x objective) covering 14.8 % (b), 21.7 % (c), 28.2 % (d), 45.8 % (e), and 60.2 % (f) of the surface. To the right, analysis of alignment efficiency for the respective matrices is shown.

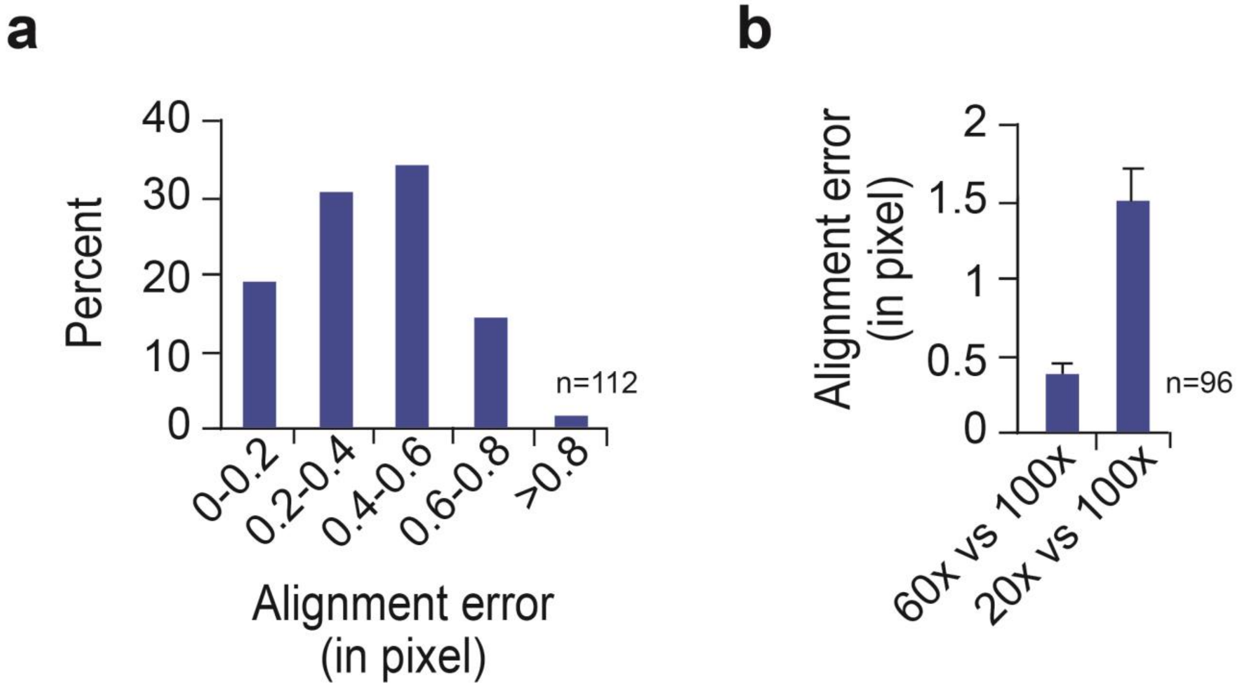

Supplementary Figure 8: Alignment Accuracy. (a) Alignment error calculated for 112 pairs of images taken from a 10,000 x 10,000 matrix with micro-pattern of random size. **(b)** Comparison of alignment error calculated for 2 images with different magnifications. To the left, alignment error using a 60x and a 100x magnification is depicted. To the right, alignment error using a 20x and a 100x magnification is shown. For both calculations, a 10,000 x 10,000 matrix with micro-pattern of random size was used.

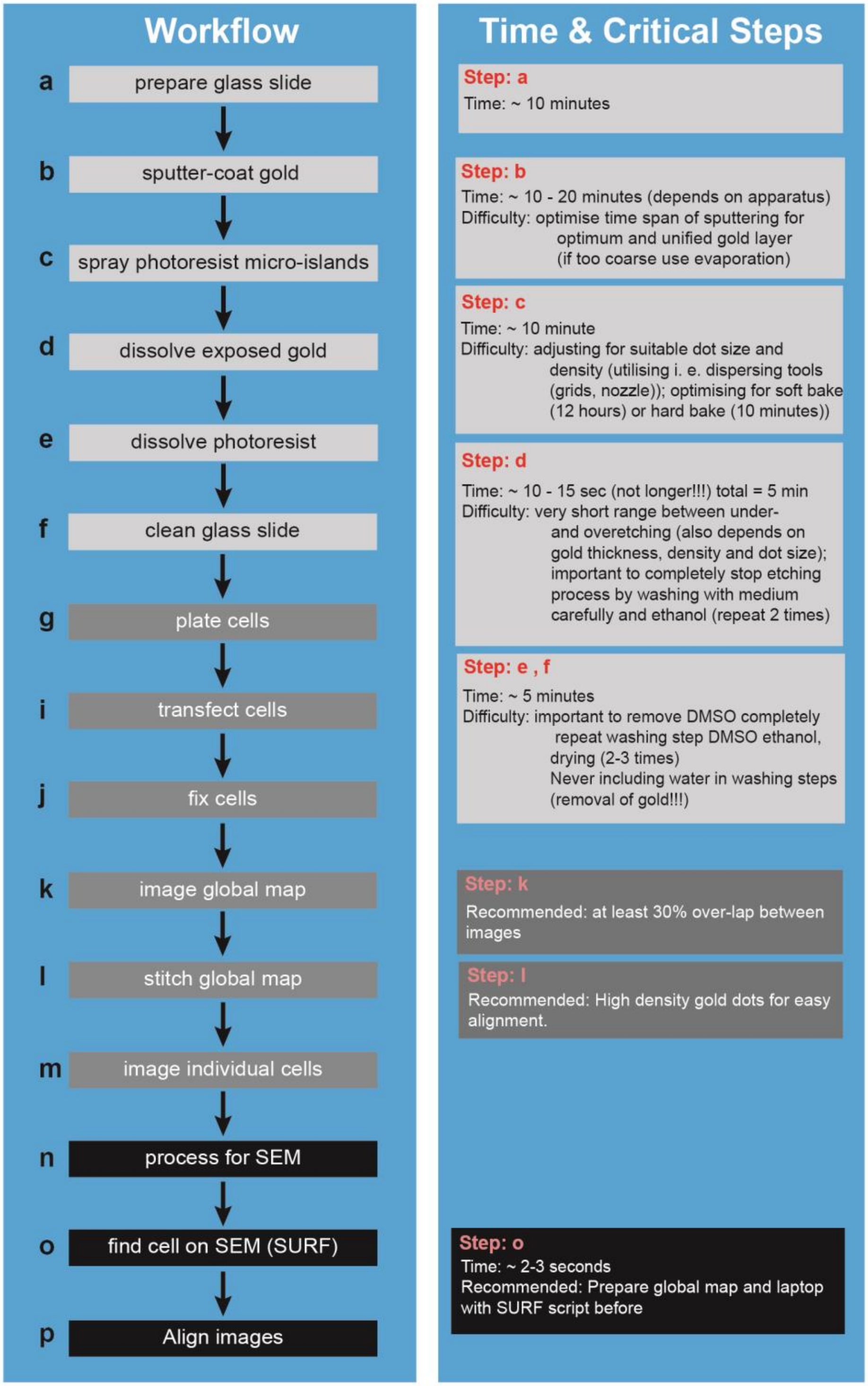

Supplementary Figure 9: Procedure overview and troubleshooting. Overview of the processing stages from gold micro-pattern preparation (light grey, a-f), cell culturing and light microscopy (dark grey, g-m) and SEM-processing (black, n-p) are shown to the left. To the right are time approximations and potential difficulties present at individual the steps.

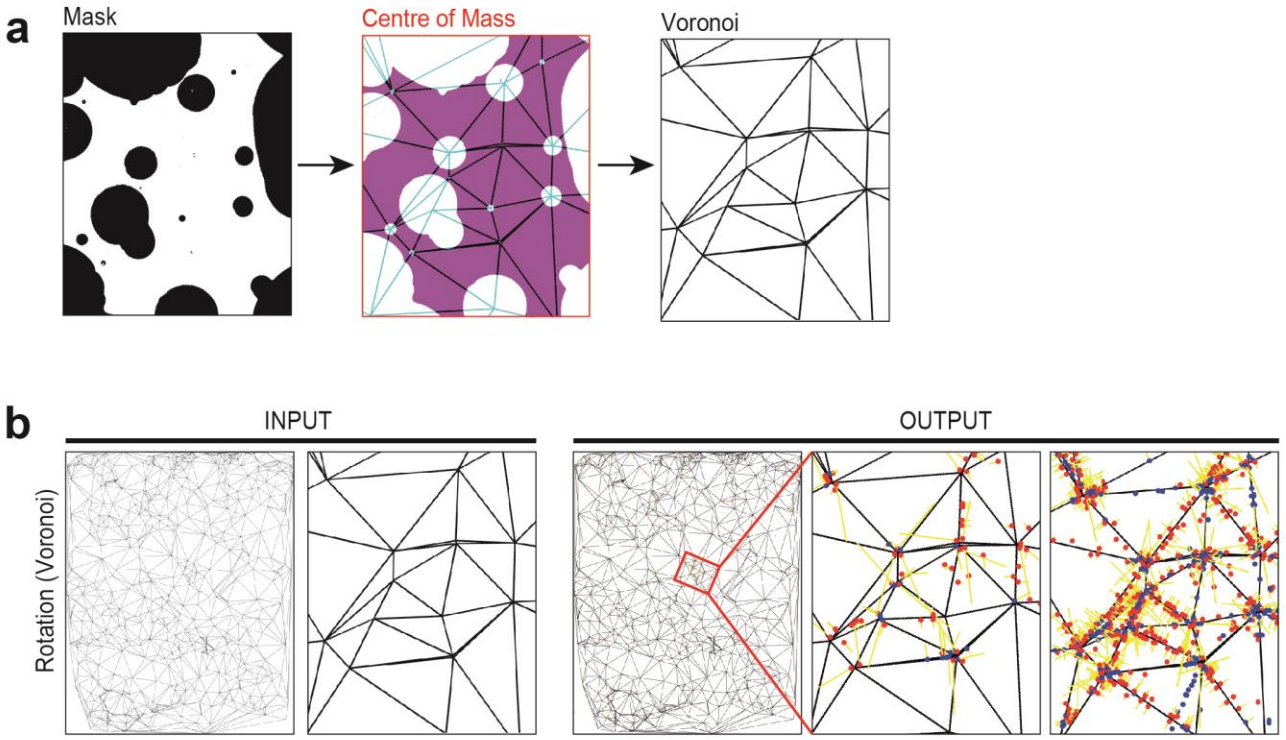

Supplementary Figure 10: Additional reference points via Voronoi polygons. **(a)** For each micro-pattern, the center of mass can be used to generate a Voronoi polygon. **(b)** Alignment of Voronoi polygons using the SURF plugin is shown. Note the additional reference points generated by the Voronoi meshwork.

SUPPLEMENTARY MOVIES

Supplementary Movie 1: Zoom into the global map coated with gold micro-patterns.

To depict the variability of gold micro-pattern in size, a 100x zoom into the global map of a 18 mm glass coverslip coated with gold micro-pattern is shown. Note that the final frame (180 μm x 180 μm) is smaller than a single image acquired with a sCMOS camera on a 60x objective (210 μm x 210 μm).

Supplementary Movie 2: Rotational alignment of micro-pattern from light and electron microscopic images via ALIGN macro.

To identify the optimal angle for image alignment, the micro-pattern from SEM back-scatter image (red) is rotated relative to the micro-pattern from the bright-field image (green). Alignment score for the relative angle is shown below.

SUPPLEMENTARY NOTE

Micro-Pattern production – time requirements and troubleshooting

All procedures for the production of gold micro-pattern were optimized for the application in correlative light-electron microscopy (**Supplementary Figure 9**). A detailed description on critical points for all steps can be found below.

In order to achieve uniform layer thickness and secure good binding of gold to the glass (**step a**), 5 minute long washing steps in acetone and ethanol are recommended.

For coating the coverslip (**step b**), any type of gold deposition can be utilized. We have tested thermal vacuum deposition and sputter-coating, and prefer the latter as it is faster. Compared to deposition by evaporation, the sputter-coated structure is supposedly coarser and slightly less homogeneous. However, we did not observe visible differences in the quality of the micro-pattern. In our hands, preparation of an approximately 25 – 30 nm thick gold layer took about 15 minutes.

For creating the later micro-pattern on the gold-coated glass coverslip (**step c**), positive photo-resist was sprayed under a shallow angle (i.e. from the side) at a distance of ~20 – 30 cm. To reduce the size-distribution of the micro-pattern, droplet dispersion can be changed using either a finer nozzle or small-meshed grids. By changing the angle, the distance or the dispersing tool, dot size range and dot density can be controlled. Note that photo-resist droplets

tend to fuse, thus creating a homogenous layer. By repeating spraying/drying cycles (~ 30 s) with small amounts of photo-resist, it is possible to create pattern with distinct dot sizes and complex shapes. Please avoid exposure to direct sunlight, as photo-resist is light sensitive.

To create gold micro-pattern (**steps d,e**), the sprayed photo-resist first has to dry onto the gold. This can be done either by drying at room temperature overnight, by soft-baking at low temperature in a heating chamber for 1-2 hours, or with a hard bake for ~10 minutes at high temperature (note: times and temperature vary between different photo-resists. See instructions of your product for details). We tested all three possibilities. While all worked, we observed that the type of drying influences its attachment strength of photo-resist to the gold layer, which is critical for the following washing procedures to remove the photo-resist (see step f below). For etching the uncovered gold areas, a diluted version of *aqua regia* was used. With increasing micro-pattern density, it is recommended to increase the amount of water in the acidic solution to prevent under-etching Note, a duration of 15 – 20 seconds should not be exceeded. Afterwards, it is important to immediately stop the etching process by washing off the acid. We find that washing in water can cause gold lift-off. In contrast, dipping the coverslip into plating medium (also to be used later on for plating cells) allowed to wash off the acid with no noticeable gold loss. After a few seconds, the etched micro-pattern coverslip was transferred into a small beaker containing ethanol and dried.

Finally, the photo-resist was removed (**step f**) by leaving the coverslips in DMSO for ~ 2 minutes. DMSO was then washed off with ethanol and let dry (~20 s). Note that left-over photo-resist affects the quality of the SEM image, but not cell growth or cell viability. To ensure that all photo-resist had been removed by DMSO, coverslips can be inspected under a light microscope: Clean micro-pattern appear as shiny particles with gold reflection, whereas micro-patterns with residual photoresist appear rainbow/greenish. As DMSO will leave streaks on the coverslip surface, repeated washing steps may be required. The micro-patterned coverslips can then be stored in a dust-free environment until use.

Before plating, the coverslips are cleaned again with ethanol, dried and put for sterilization 10 minutes under UV-light. Generally, the micro-pattern should not get into contact with pure water as this may cause lift-off of gold dots. Further, care should be taken in terms of scratching the surface with forceps during handling (an example can be found in **Supplementary Figure 4c,** panel g). During plating and imaging no additional care is necessary.
